# Stress and Burden Experienced by Parents of Children with Type 1 Diabetes—A Qualitative Content Analysis Interview Study

**DOI:** 10.3390/children12080984

**Published:** 2025-07-26

**Authors:** Åsa Carlsund, Sara Olsson, Åsa Hörnsten

**Affiliations:** Department of Nursing, Umeå University, 901 87 Umeå, Sweden; sara.olsson@umu.se (S.O.);

**Keywords:** children, parents, qualitative content analysis, type 1 diabetes

## Abstract

**Highlights:**

**What are the main findings?**

- The complex factors of stress and burden in parenting a child with T1D can be like tying oneself in knots.
- Constant stress and worry lead to parental exhaustion, sleeplessness, and cognitive issues, the balance between fostering independence and diabetes management adds to these challenges.

**What is the implication of the main finding?**

- Fund and incorporate social workers and psychologists with expertise in T1D into standard diabetes care teams.
- Include psychosocial support as a standard part of care, with scheduled visits for parents.

**Abstract:**

**Background:** Parents of children with type 1 diabetes play a key role in managing their child’s self-management, which can be stressful and burdensome. High involvement can lead to reactions such as emotional, cognitive, and physical exhaustion in parents. Understanding parents’ psychosocial impact due to their child’s disease is crucial for the family’s overall well-being. The purpose of this study was to describe stress and burden experienced by parents in families with children living with type 1 diabetes. **Methods:** This study utilized a qualitative approach, analyzing interviews with 16 parents of children aged 10 to 17 years living with T1D through qualitative content analysis. The data collection occurred between January and February 2025. **Results:** Managing a child’s Type 1 diabetes can be tough on family relationships, affecting how partners interact, intimacy, and sibling relationships. The constant stress and worry might leave parents feeling exhausted, unable to sleep, and struggling to think clearly, on top of the pain of losing a normal everyday life. The delicate balance between allowing a child with type 1 diabetes to be independent and maintaining control over their self-management renders these challenges even more demanding for the parents. **Conclusions:** Parents’ experiences highlight the need for robust support systems, including dependable school environments, trustworthy technical devices, reliable family and friends, and accessible healthcare guidance. These elements are essential not only for the child’s health and well-being but also for alleviating the emotional and practical burdens parents face.

## 1. Background

Type 1 diabetes (T1D) is a lifelong autoimmune disease, treated with insulin injections via pen or pump. The etiology of T1D is not entirely understood, but genetic factors, environmental influences, and certain viruses appear to play a role [1,2,3]. In Sweden, 8712 children (17 years and under) currently live with T1D. Although the number has risen, it has plateaued in children and adolescents [4]. The self-management of T1D involves a complex combination of insulin administration, blood glucose monitoring, carbohydrate counting, sleep, exercise, problem-solving, reducing risks, and healthy coping [5,6]. Over the past decade, several technological advancements have been made, such as continuous glucose monitoring, sensors, alarms, and insulin pumps with continuous and closed-loop-systems for insulin delivery. Despite these advancements, childhood and adolescence can be extremely stressful for families of children with T1D, and previous studies show psychosocial support from grandparents, siblings, and friends is of great importance [7,8]. The ongoing stress may affect the family climate in both the short and long term [9,10]. Previous research has found that mothers suffer from severe symptoms of distress [11,12,13], and in contrast, fathers are more likely to experience anxiety [14,15]. However, there is limited research on fathers’ psychological symptoms in the context of T1D [16], as well as the challenges faced by other close family members [6,17]. Furthermore, there are associations between frequent family conflicts and poor adherence to treatment and well-being in children with T1D [6,8,18,19], which in turn might negatively impact stress [6,19] and burden [20] in parents.

Parents are fundamental to their children’s development [21]. Despite its significance, parenting remains a challenging and demanding responsibility [22]. Parents of healthy children experience typical yet occasionally stressful and demanding phases, such as the child’s hormonal fluctuations and a growing sense of autonomy. These phases can be even more challenging as children gain independence while managing T1D [23,24,25,26]. This may lead to parental stress [27,28] and burden [20,29]. Stress concerns emotional and psychological challenges, such as fear of hypo- and hyperglycemia, carbohydrate counting, technical aids, and long-term complications [15,30]. Burden includes the overall impact on various aspects of the parents’ lives, i.e., time, energy, lack of support and social isolation [29,31], and both can significantly affect the well-being of parents and their ability to care for their child effectively.

In summary, parents of children with T1D play a crucial role in managing their child’s self-management, which can be both stressful and burdensome. This high level of involvement can lead to stress reactions, marked by emotional, cognitive, and physical exhaustion in parents [32,33]. Understanding how parents are psychosocially affected by their child’s T1D is crucial for the entire family’s short- and long-term well-being [14,15,27].

## 2. Methods

### 2.1. Aim

The aim of this study was to describe stress and burden experienced by parents of a child with T1D.

### 2.2. Design

This study used a qualitative approach, reporting results from interviews of parents of children (10–17 years) living with T1D analyzed with qualitative content analysis [34,35].

### 2.3. Participants

Sixteen parents from Sweden, consisting of fourteen mothers and two fathers, who met the inclusion criteria of having a child aged 10 to 17 with T1D (mean age 13.5 years). Table 1 describes the background of the participants, with ages ranging from 40 to 62 years (mean age, 48). Thirteen had a university education, while three had a high school education. Eleven participants were married or cohabiting, and five were divorced. Four participants lived in urban areas, and twelve in rural areas.

### 2.4. Data Collection

The participants were recruited through convenience sampling within an ongoing research program—a digital survey study in which prospective participants were asked to provide their contact information if they agreed to be contacted for future studies. Before deciding to participate in this study, all participants received oral and written information about the aims and procedures and were recruited with informed consent. The data collection lasted from January to February 2025. The individual interviews lasted between 54 and 76 min and were recorded using Microsoft Teams software, because visual recording captures non-verbal cues and contextual details that might otherwise be overlooked. An interview guide with background questions and open-ended questions was used. All the questions started with “can or would you like to tell me about”, the questions asked were, “… describe a typical day for you as a parent with a child living with T1D, … the kinds of worries or stress you’ve experienced since your child was diagnosed with T1D, …situations that feels especially stressful, …times when you’ve felt alone in managing your child’s diabetes, … the one you usually turn to for support, … your relationship with your family members since the diagnosis, your family’s social life since your child was diagnosed, …your hopes and concerns for your child’s future living with T1D. We initially believed we had gathered enough data to identify key differences in experiences [34] by the fourteenth interview; however, we conducted two more interviews to see if additional data would improve our understanding. It was concluded that these additional interviews did not enhance our comprehension of the issue. Thereafter, the authors transcribed the recordings into text.

### 2.5. Analysis

To enhance the understanding of parents’ experiences of living with a child with T1D, the interviews were analyzed using the hands-on qualitative content analysis guide outlined by [34,35]. The first step was the de-contextualization phase, during which the transcribed text was read multiple times to gain a general understanding before being divided into meaning units. These units were then condensed and labeled with a code to retain their essential content. In the re-contextualization phase, the codes were compared and organized into groups based on their similarities and differences. The analysis continued with the goal of organizing the text into preliminary subthemes and themes based on interpretation and abstraction. The analysis was a dynamic process with movements back and forth between transcribed interviews, and levels of interpretation and abstraction.

### 2.6. Trustworthiness

To ensure trustworthiness, we reflected on credibility, dependability, and transferability of our method. Qualitative content analysis was used to analyze the interviews because it is a structured method that suited the aim of the study with purpose to describe variations in experiences [34,35]. To ensure the data was thoroughly described and categorized while maintaining the integrity of the parents’ responses, de-contextualization and re-contextualization were performed. Credibility was enhanced by presenting representative quotations from the transcribed text, allowing readers to see direct examples of the participants’ experiences, as well as disconfirming cases which further encouraged the variation in experiences. The researchers have personal experiences of living with T1D or T2D, and the preunderstandings were discussed during the process to make sure the participants voices were reported in the results. However, the interpretations are always co-creations between the participants and the researchers. Furthermore, agreement among the co-researchers ensured that the interpretations were presenting the most probable meanings of the participants’ perspectives in display. The study’s ethical considerations, including adherence to the Helsinki Declaration and approval by the Swedish Ethical Review Authority, further supported the credibility of the research. Descriptions of the participants’ characteristics and the data collection and analysis process were provided to facilitate transferability. Presenting the findings along with quotations allows readers to judge the applicability of the findings to other contexts.

### 2.7. Ethical Considerations

The ethical guidelines of the Helsinki Declaration and the World Medical Association were followed, and the ethical application for the study was approved by the Swedish Ethical Review Authority (Dno: 2024-04062-01). Participation was voluntary. Written informed consent was obtained from all participants and efforts were made to protect the participants’ integrity throughout the entire process. Each consent was marked with a unique digit assigned to each participant, and these unique digits were retained during all stages of the research process.

### 2.8. Results

Being a parent to a child with T1D related to stress and burden was interpreted as the parents tying themselves in knots, presenting the main theme of the results. The parents experienced relational challenges in family dynamics, with struggles in partner interactions and intimacy, sadness about overlooked siblings, and a fine line between independence and control. Further, the parents described years of exhaustion, sleeplessness and fatigue, grief over a lost “normal” life, and overwhelming lifelong responsibilities. They also struggled to find reliable support channels, emphasizing the importance of dependable school environments, the need for trustworthy technical devices, reliable backups for assistance when necessary, and accessible healthcare guidance. The analysis revealed four themes with 3–4 subthemes each. These are described in Table 2 below and are more detailed in the text, where quotations are given as examples of the subthemes to increase understanding.

## 3. Tying Oneself in Knots

The main theme of the results was that parents tied themselves in knots managing the variation in complex factors related to stress and burden connected to managing T1D. The knots were illustrated to get tied harder and harder, as a result of years of exhaustion related to being a parent to a child with T1D. Related to relational challenges in family dynamics, the threads were interpreted to get intertwined when family members pulled them in different directions. Related to the search for resilient support systems, the parents asked for help to loosen the ties and to find coping strategies in their emotional, psychosocial, and practical tangled up everyday life.

### 3.1. Years of Exhaustion and Pressure

Stress and burden experienced by parents managing a child’s T1D was described as years of exhaustion for many parents. They experienced sleeplessness and fatigue due to the constant need to monitor their child’s blood sugar levels, which led to cognitive difficulties and impaired concentration. The grief over a lost normal life was profound as parents navigate the emotional burden of their child’s disease. Furthermore, the overwhelming lifelong responsibilities of managing T1D resulted in feelings of isolation and despair.

#### 3.1.1. Sleeplessness and Fatigue

Many parents reported significant cognitive difficulties due to chronic sleep deprivation and fatigue. The constant need to monitor their child’s blood sugar levels and respond to alarms disrupted their sleep patterns, leading to poor memory and difficulty concentrating. Several parents chose to sleep in their child’s room or bed to avoid frequent nighttime disruptions. This ongoing necessity to monitor and care for their child resulted in a type of fatigue that many parents described as unique.

The cognitive strain affected work and activities, with parents struggling to focus and stay productive due to exhaustion from sleeplessness. Chronic fatigue significantly impacted their lives, as constant vigilance to manage their child’s condition left them exhausted, impairing cognition and well-being.

“Yes, but you live in five-minute intervals in a way. I haven’t had the energy to read books for a long time. That feeling of lying down with a fiction book and enjoying it… Instead, it can be like this: after 5 min, I must stop to check, so there are constant distractions. It’s a huge distraction, and it’s hard to focus. I talk to myself sometimes and say, can’t you ever finish anything?” (P14)

#### 3.1.2. Grief over a Lost “Normal” Life

Parents experienced deep grief that was multifaceted, including the loss of a “normal” life, constant worry about their child’s health, and the emotional burden of managing a lifelong illness. The realization of how serious the condition was brought feelings of vulnerability, sadness, and fear. At first, most parents felt shocked, overwhelmed, and emotionally drained. The grief was ongoing as they faced new challenges and uncertainties continuously. This lasting sorrow often caused feelings of hopelessness and despair, negatively impacting their mental health and overall outlook on life. Parents often struggled to find joy in everyday activities and felt life had become just a series of tasks to do for their children.

The emotional impact of managing a lifelong illness like T1D was significant. Parents had to navigate the complexities of their child’s condition while handling their own emotional responses. This ongoing grief affected their ability to fully participate in life, both personally and professionally. The constant worry and emotional burden led to a sense of diminished quality of life.

“I had to keep living for my children’s sake, but I didn’t think it was fun to be alive. I felt in the evenings, that it was good to have another day done.” (P06)

#### 3.1.3. Overwhelming Lifelong Responsibilities

The parents faced overwhelming lifelong responsibilities. The emotional burden of managing T1D significantly affected them. Many often felt overwhelmed by the responsibility of keeping their child safe and experienced a range of emotions, including fear of hypoglycemia (low blood sugar), frustration with the unpredictability of blood sugar levels, and sadness about their child’s condition. These feelings sometimes led to helplessness and despair. Several parents felt isolated and unsupported because few people truly understood the complexities of the disease. The ongoing worry about their child’s health and the risk of life-threatening complications added to their emotional strain. Several parents stated they must remain strong and resourceful to manage the challenges of caring for their child’s condition T1D, sometimes with endlessness and exhaustion.

“What happens if I get sick and die? I can’t. I must live until he’s 50. Well, it’s not that fun to think about.” (P14)

They developed various coping strategies to manage the lifelong challenges associated with T1D, including seeking psychological support, establishing routines, and building social support networks. However, the need for continuous adaptation and problem-solving remained a constant source of stress.

“To always be one step ahead takes energy, or for me, it takes a lot of energy, that is very heavy, yet I don’t show it directly to others.” (P21)

### 3.2. Relational Challenges in Family Dynamics

The pressure experienced by parents managing a child’s T1D brought numerous relational challenges in family dynamics. Parents often struggled with partner interactions and intimacy. They also experienced sadness about overlooked siblings, who may not have received the attention they deserved. Additionally, parents faced the fine line between fostering their child’s independence and maintaining control over diabetes management.

#### 3.2.1. Struggles in Partner Interactions and Intimacy

The parents struggled with partner interactions and intimacy, affected by their child’s life-threatening illness. Stress and worry led to misunderstandings and arguments, creating feelings of overwhelm and frustration. The constant vigilance, especially at night, caused exhaustion, reducing time and energy for each other. This fostered emotional distance and isolation.

Some parents eased nighttime anxiety by sharing a bed or room with the child. Others, exhausted, chose one parent to care for the child at night while the other slept separately, often without the sensor alarm. One parent shared that their mood was very unstable initially, sometimes sleeping at 6:30 p.m. after a sleepless night, lacking motivation for closeness. Because he couldn’t communicate and slept in a different room, the partner was unaware of his struggles.

“We stopped being intimate, we didn’t sleep together, then… you grow apart, you’re not that close anymore, and then, I don’t know… our entire lives were about our child’s ups and downs, keeping it as straight as possible” (P22)

Parents revealed working tirelessly night and day behind the scenes, managing the child’s fluctuating blood sugar levels and handling nighttime responsibilities, planning, cooking, and maintaining continuous contact with the school and healthcare providers. While most parents shared the burden and supported each other, a few parents voiced disappointment in their partner or ex-partner. A parent expressed disappointment in their partner for not being involved in managing their child’s T1D but noted that the partner accepted all the praise from healthcare professionals, family, and friends. Along with disappointment and resignation, the strain led to psychological burnout and sickness leave for the parent in question.

“I have to fight and pull them and push and all that and then when something goes well and we meet the doctor or friends then he talks as if everything is going well without my effort and I’m really bitter about that and feel like he takes credit for everything. It’s not that he doesn’t have an intention or will or care. He does, but he has like zero ability for structure” (P9)

Parents sometimes expressed that they bore the heaviest responsibility for their child’s care. One parent mentioned that discussions about sharing parental responsibilities more equally often led to disagreements, stagnation, and feelings of loneliness. Additionally, another parent recounted that their ex-partner appeared unimpressed by their child’s high blood sugar levels. This indifference caused disappointment and widened the gap between them. Some parents also noted that their partners mainly focused on managing their child’s low glucose levels and less frequently addressed high glucose levels.

#### 3.2.2. Sadness About Overlooked Siblings

Parents expressed sadness about overlooked siblings. Siblings were involved in demanding situations, but they should not have to bear the burden of T1D. The parents had not placed responsibility on the siblings because they felt it was not their duty to be responsible for someone else’s life. However, many parents felt guilty about the siblings and believed they deserved more attention and care. The parents expressed sorrow and grief for the siblings, stating that they were often overlooked and deserved to be seen and heard. They struggled with managing the needs of all children in the family, balancing guilt for the siblings with the unconditional self-management requirements of their child’s T1D. Some parents disclosed that, in certain situations, when siblings sought their attention, they did not have time for them. In some families, siblings withdrew from other family members and maintained distance, such as limiting communication and interactions. In other families, siblings grew closer and supported each other, providing emotional support or helping with practical tasks like household chores or caring for a younger sibling. A parent described how a younger sibling tried to help when parents were occupied due to the demands of T1D.

“I come upstairs and there my five-year-old is standing, undressed, with her clothes folded in a pile. She has put on a nightgown, she has brushed her hair and is brushing her teeth, she says: I understand Mom that when you have a child with diabetes, siblings must take care of themselves because Mom and Dad don’t have time” (P69)

#### 3.2.3. A Fine Line Between Independence and Control

Parents struggled to balance independence and control as their child with T1D grew older. They aimed to allow decision-making while ensuring effective diabetes management, which was complicated by hormonal changes during adolescence. Blood sugar levels were unpredictable, often causing conflicts when children wanted to spend time with friends or online, or during exercise. Some parents also worried about their child’s social and educational challenges and their ability to manage the condition independently.

### 3.3. Search for Resilient Support Channels

The parents searched for resilient support channels. Parents managing a child’s T1D stressed the importance of a dependable school environment, trustworthy technical devices, reliable support from family and friends, and accessible healthcare guidance. These support systems were seen as essential for ensuring their child’s health and well-being, while also relieving the emotional and practical burdens faced by parents.

#### 3.3.1. Importance of Dependable School Environment

Parents felt frustrated by the school’s lack of support, as many had not received necessary professional resources for their children and believed the schools were out of funding. They didn’t know who to turn to and thought it should be obvious that a small child with T1D needs dedicated resources. One parent said it took a long time to understand the rules and contacts, showing a lack of clear communication from school staff. Another parent noted the principal missed a crucial meeting about their child’s resources, leaving parents feeling anxious and unsupported.

They revealed their children had high blood glucose and nervousness before tests, increasing parents’ stress. Initially trusting the school to manage their child’s condition, their view changed after incidents where no one helped during low blood glucose episodes, such as in swimming lessons. They described the school situation as unsustainable or dangerous, citing a lack of understanding of T1D and unengaged or arrogant staff, which added stress. One parent said professionals often don’t know what to do, leaving parents to guide them.

“I am always stressed and, in a hurry, knowing that the school is accountable for three years, while I am responsible for his entire life.” (P03)

Parents stressed trusting school professionals to manage T1D effectively. Schools with knowledgeable staff and self-management resources could predict and handle low blood sugar, allowing children to participate fully. Proactive schools that collaborated with parents fostered a supportive, reassuring environment. A parent noted regular updates from the principal kept everyone informed, creating a supportive atmosphere. Consistent communication and routines provided security and fostered independence, with parents involved when necessary.

Most parents were mainly concerned about their child’s health and safety at school, as the disease could affect cognitive performance. Proper T1D management by school staff could prevent educational disruptions and promote a positive experience. Parents appreciated attentive, responsive staff that created a safe environment. Collaboration between parents and staff was essential to ensure care, support, and minimize the disease’s impact on academic and social life.

#### 3.3.2. Need for Trustworthy Technical Systems

Parents explained that T1D self-management technology, such as continuous glucose monitors (CGMs) and insulin pumps, provides valuable data and support. However, they also expressed concerns about the reliability of these devices. Technical issues, sensor failures, and data inaccuracies could add to their stress and anxiety. These technological devices are seen as beneficial when working properly, but malfunctions could lead to disrupted nights with sensor transfers and troubleshooting, and even days when parents have to visit their child’s school to resolve issues. Some parents do not fully rely on technology and remain cautious. They view technology as a useful tool but emphasize the need for a backup plan and a safety system that meets their family’s needs. This cautious approach comes from understanding that technology, while helpful, is not infallible and requires ongoing monitoring and intervention.

#### 3.3.3. Reliable Backups for Assistance When Necessary

The parents emphasized having reliable backups for support when needed. Support from the extended family and close friends was invaluable. These networks provided both emotional support and practical assistance, such as monitoring blood sugar levels and administering insulin when needed. The close-knit family ties and friendships provided a sense of security and reduced feelings of isolation associated with caregiving responsibilities.

The initial shock of the diagnosis could be overwhelming, with parents constantly worrying about their child’s health. The calm presence of a supportive family member or friend was a lifesaver, providing reassurance and practical help. This support allowed parents to share the burden and find moments of respite, knowing that their child was in capable hands.

“You feel so shocked at first that you can’t even sleep because you’re constantly thinking. My child will die if I am not awake and keep an eye on his blood sugar; that is just the beginning. My mother has always remained calm, and I think that has saved us. Plus, my fantastic colleague who always listens and gives me great advice” (P02)

Some grandparents stopped babysitting after the T1D diagnosis due to concerns about monitoring blood glucose and managing emergencies, especially if unfamiliar with the latest technology. This lack of confidence led to stepping back, disappointing parents and the child. Parents felt frustrated yet understanding of their parents’ fears but also isolated without support. They noted some grandparents showed disinterest, which was discouraging, increasing their sense of being overwhelmed and longing for more involvement. The absence of reliable support heightened their stress disappointment.

#### 3.3.4. Wish for Reachable Healthcare Guidance

Several parents expressed a desire for more structured support from healthcare providers, such as regularly scheduled counseling sessions, ideally coordinated with their child’s medical appointments. Parents showed a preference for automatic and standardized standards of care for psychological support, especially when they were hesitant to seek help on their own. The initial shock of the diagnosis could be overwhelming, and ongoing psychological support was essential for both parents and children to handle the emotional and practical challenges of managing T1D.

“We had a counselor’s talk when we were in the hospital, but it was such a shock back then… I wish we had regular appointments later, both my husband and I need them, but we can’t bring ourselves to organize it” (P22)

Parents reported gaps in the healthcare system, particularly in guidance related to hormonal changes and diet management. A few parents felt that healthcare providers lacked the necessary knowledge and flexibility to support their child’s unique needs, such as the impact of hormonal changes on blood sugar levels. This lack of comprehensive support left parents feeling unsure of how to manage their child’s condition best.

## 4. Discussion

Our results broadly showed that managing a child’s T1D brings relational challenges, feelings of stress, exhaustion, sleeplessness, cognitive difficulties, and profound grief over lost normalcy in everyday life. Essential support systems included dependable schools, trustworthy technical devices, reliable family and friends, and accessible healthcare guidance.

In summary, the main theme of the study was illustrated as parents tying themselves in knots, managing the multifaceted factors related to stress and burden being a parent to a child living with T1D. The knots were tightened after years of exhaustion and the first theme of the results described the impacts at an internal level. The second theme involved relational challenges in the family dynamics, where loose ends were pulled in different directions and were described with impacts on a family level. The last theme illustrated the importance of resilient support systems, and this was connected to further outer levels, such as organizational and societal. According to Bronfenbrenner [36], the child’s relationship with other family members changes as the child ages. In the event of serious events, such as a T1D diagnosis, the relationships of the family system change. The family becomes involved in a dynamic interaction with the surrounding society and the child’s development is affected in different ways by these.

Our findings implied that parents’ management of T1D in a child impacts several relational dynamics. In line with [37], our results indicated that intimate relationships could become strained as parents navigate the stress and demands of diabetes management, which can disrupt routines and family roles. This aligns with Lazarus and Folkman’s Transactional Model of Stress and Coping [38], which views stress as a dynamic process resulting from the interaction between an individual and their environment. According to this model, stress occurs when individuals see that the demands of a situation exceed their coping resources. In the context of managing a child’s Type 1 Diabetes (T1D), parents may judge the situation as highly stressful, especially when routines are disrupted, and traditional family roles are challenged. This perception can cause emotional strain and conflict within close relationships. The model also highlights the importance of coping strategies—both problem-focused and emotion-focused—in influencing the impact of stress. Additionally, our findings, along with those of [38], showed that as spouses support each other more over time, they experience fewer conflicts during discussions about their child’s T1D. This mutual support serves as a form of emotion-focused coping [38], enabling partners to handle the emotional strain of caregiving better. It also promotes a sense of shared responsibility and understanding, which leads to a healthier relationship dynamic.

The results highlighted the need for processing existential factors related being a parent to a child living with T1D, such as connected to despair with an impact on the overall outlook on life and thoughts on what would happen to the child if the parent died. The international Society for Pediatric and Adolescent Diabetes [ISPAD] and de Wit et al. [39] highlight that psychological parental support is essential in child and adolescent diabetes care, such as training in coping strategies. Parental support can with advantage, be organized by healthcare with group discussions [40], where parents can share their emotions and receive peer support. Peer support groups were not mentioned in our results, which could be due to several reasons. It is possible that these groups are not easily available or accessible to parents of children with T1D. Additionally, parents may be too overwhelmed by the practical and emotional burdens of managing their child’s condition to seek out or participate in these groups actively, or there may be a lack of awareness about the existence and benefits of peer support groups [34].

In line with our results, Overgaard et al. [41] highlighted that siblings frequently felt misunderstood and dissatisfied with their T1D-related family role, even though they emphasized wanting to be more actively involved in their sibling’s diabetes [42]. Our results imply that some grandparents stop watching their grandchild due to concerns about managing T1D, leading to a lack of family support and an increased burden on the parents. This is supported by Zhang et al. [43], who also report that parents suffer from a lack of understanding from relatives.

Our results show that many parents express worries about the commitment and level of education of school staff. These concerns seemed to arise when there was a lack of clear and open communication systems. Previous research showed that active training and structured education for school professionals are crucial and empowering, leading to increased staff confidence in managing the daily needs and possible emergencies of school children with T1D [44,45].

Moreover, the results show that parents of children with T1D face both emotional and practical challenges [46,47]. In line with other studies [48,49], our results indicate that the constant awareness and need to monitor blood sugar levels continuously disrupt parents’ personal and professional lives in a way that is often unnoticed by others, typically leading to ongoing stress and sleep deprivation [37,50]. A parent in this study referred to it as working behind the scenes. This, in turn, impacted cognitive functions, work performance, and the ability to engage in leisure activities [51]. The T1D diagnosis brought a profound sense of grief and loss, as parents’ express grief in the loss of a “normal” life and struggle with the ongoing emotional burden of managing T1D [52]. The responsibility of ensuring their child’s safety can be overwhelming, causing feelings of isolation and helplessness, even though the majority of parents’ priority is to keep their child healthy and safe [53,54]. Parents must balance their child’s growing need for independence with the necessity of managing diabetes effectively [30], which can lead to conflicts and additional stress [55,56].

Furthermore, parents noted that T1D management technologies, such as continuous glucose monitors (CGMs) and insulin pumps, offer valuable insights and assistance [49,57]. However, they had concerns about the reliability of these devices. Technical malfunctions, sensor issues, and data inaccuracies increased stress and anxiety. Some parents could not fully depend on technologies and had to remain vigilant [58,59]. Parents emphasized the importance of support from extended family and friends [60,61]. Several parents would like more organized support from healthcare services, such as regularly scheduled counseling aligned with their child’s medical office visits. They prefer automatic psychological support offers, acknowledging they might hesitate to seek help independently [62,63]. Some parents cite deficiencies in the healthcare system, particularly regarding guidance on hormonal changes and dietary management [59].

### 4.1. Limitations

One limitation of the study was that the participants were predominantly women, which may have influenced the results. It is possible that men’s and women’s experiences differ. However, to investigate this was not the aim of the study. A further limitation concerning the participants was their equivalent cultural backgrounds, only one parent was born in a different country than Sweden. The study utilized convenience sampling, which is a common and efficient method in qualitative research. However, it is important to acknowledge that this approach might not fully capture the range of parental experiences, as it depends on an easily accessible sample instead of a representative one of the entire population. Including parents of children with T1D aged 10 to 17 in the study offers both advantages and disadvantages. On one side, it captures a variety of perspectives and developmental stages, providing a more comprehensive view of the challenges faced by parents. This broader inclusion can also make the results more relevant to a wider group of parents with children of different ages. However, the wide age range might introduce variability in the data, as parents’ needs and experiences can differ significantly based on their child’s age.

### 4.2. Conclusions

Managing a child’s T1D presents profound relational challenges within families, impacting partner interactions, intimacy, and sibling dynamics. The relentless stress and worry lead to parental exhaustion, sleeplessness, and cognitive difficulties, compounded by the grief over a lost everyday life. The delicate balance between fostering independence and maintaining control over diabetes management further intensifies these challenges. The multifaceted factors related to stress and burden being a parent to a child living with T1D can be illustrated as tying themselves in knots. Parents’ experiences highlight the critical need for robust support systems, including dependable school environments, trustworthy technical devices, reliable family and friends, and accessible healthcare guidance. These elements are essential not only for the child’s health and well-being but also for alleviating the emotional and practical burdens parents face. Addressing these needs comprehensively can significantly improve the quality of life for families navigating the complexities of T1D management. To meet these needs, we suggest the following:

Healthcare Systems

- Integrate social workers and psychologists with expertise in chronic illness and family systems into standard diabetes care teams.
- Include psychosocial support as a routine component of care, with scheduled visits for parents to address emotional strain, relationship challenges, and coping strategies.

Policy Makers

- Allocate funding for long-term psychosocial support programs for families affected by T1D.
- Advocate for family-friendly workplace policies, such as flexible hours and caregiving leave, to support parents managing T1D.

Finally, while this study contributes to the growing body of research on family dynamics in T1D, it also highlights significant gaps in the literature. There remains a lack of qualitative studies that explore the lived experiences of families, particularly how they emotionally and relationally adjust to the ongoing demands of T1D. Additionally, few studies adopt longitudinal or systemic perspectives, limiting our understanding of how family roles, coping strategies, and relationships evolve over time. Addressing these gaps is essential for developing more effective, empathetic, and sustainable support systems for families navigating the complexities of T1D.

## Figures and Tables

**Table 1:** Description of the participants.

Age range (m)	40–62 (48)
Mother Father	*n* = 14 *n* = 2
Education High School University	*n* = 3 *n* = 13
Marital status Married/Cohabiting Divorced	*n* = 11 *n* = 5
Place of living Rural Urban	*n* = 12 *n* = 4

**Table 2:** Main theme, themes, and subthemes describing the stress and burden by parents of a child with T1D.

Main Theme	Themes	Subthemes
Tying oneself in knots	Years of exhaustion and pressure	Sleeplessness and fatigue Grief over a lost “normal” life Overwhelming lifelong responsibilities
	Relational challenges in family dynamics	Struggles in partner interactions and intimacy Sadness about overlooked siblings A fine line between independence and control
	Importance of resilient support channels	Importance of dependable school environments Need for trustworthy technical systems Reliable backups for assistance when necessary Wish for accessible healthcare guidance

## Data Availability

The data that support the findings of this study are not openly available due to reasons of sensitivity and are available from the corresponding author upon reasonable request.
